# Integration of mental health services with HIV prevention, treatment and care

**DOI:** 10.2471/BLT.25.293646

**Published:** 2025-09-03

**Authors:** Erin K Ferenchick, Zeinab Hijazi, Anurita Bains, Savvy K Brar, Ani Shakarishvili, Paul Bolton, Neerja Chowdhary, Tarun Dua, Jerome Galea, Yves Miel Zuniga, Gavin Reid, Scott Laurence Chiossi

**Affiliations:** a Health Systems Team, United for Global Mental Health, C/O Kreston Reeves Unit 2, 168 Shoreditch High Street, E1 6RA, London, England.; b Global Mental Health Leadership Team, United Nations Children's Fund, New York, United States of America (USA).; c Global HIV-Team, United Nations Children’s Fund, New York, USA.; d Division of Data, Analytics, Planning and Monitoring, United Nations Children’s Fund, New York, USA.; e Office of Deputy Executive Director – Programme, Joint United Nations Programme on HIV/AIDS, Geneva, Switzerland.; f Department of Mental Health, Johns Hopkins University Bloomberg School of Public Health, Baltimore, USA.; g Department of Mental Health, Brain Health and Substance Use, World Health Organization, Geneva, Switzerland.; h School of Social Work, University of South Florida, Tampa, USA.; i Community, Rights and Gender Department, The Global Fund to Fights AIDS, Tuberculosis and Malaria, Geneva, Switzerland.; j United Nations Inter-Agency Task Force on the Prevention and Control of Noncommunicable Diseases, World Health Organization, Geneva, Switzerland.

Given the current landscape of service disruptions and funding challenges, it is critical to ensure that the integration of mental health and human immunodeficiency virus (HIV) services remains a high priority. Doing so is essential as countries strive to reach the 2030 global treatment and prevention targets for achieving HIV epidemic control. Strong evidence connects mental health comorbidities, such as depression and anxiety, to poor outcomes along the HIV care continuum by being particularly detrimental to prevention, retention in care, adherence to antiretroviral therapy and viral suppression.^1^^–^^3^ Estimates from United for Global Mental Health’s report suggest that integrating mental health into HIV prevention initiatives could hasten the decline in new HIV infections by at least 10.0% and as much as 16.5%, meaning that more than 924 000 people could avoid acquiring HIV during the decade from 2020 to 2030, based on the 2000–2019 data from the Joint United Nations Programme on HIV/AIDS (UNAIDS).^4^

Several population groups are especially vulnerable, underscoring the need for service integration. Adolescents and young adults living with HIV often face stigma, discrimination and mental health challenges such as depression and anxiety.^5^ Children and adolescents living in households where a caregiver is living with HIV face unique challenges, including fear, stigma and anxiety. All these factors also have broader effects on family dynamics and emotional well-being of young people. Other vulnerable groups include key and priority populations such as men who have sex with men, trans and gender-diverse people, sex workers, people who inject drugs, prisoners and people living with HIV aged 50 years and older, who are disproportionately affected by a double burden of HIV and mental health conditions.^6^

The global HIV community has been focusing on integrating mental health and psychosocial support into its programmes since the early stages of the HIV response. Current global guidance takes existing evidence into consideration and provides valuable frameworks for integrating the full continuum of mental health care. Available guidance includes key documents such as the UNAIDS and World Health Organization’s (WHO) *Integration of mental health and HIV interventions – key considerations*; WHO’s *Mental Health Gap Action Programme (mhGAP) guideline for mental, neurological and substance use disorders*; the United Nations Children’s Fund’s (UNICEF’s) *Global multisectoral operational framework for mental health and psychosocial support of children, adolescents and caregivers across settings*; WHO and UNICEF’s *Guidelines on mental health promotive and preventive interventions for adolescents: helping adolescents thrive* and related toolkit; and WHO and UNICEF’s *Mental health of children and young people: service guidance*. Despite this overwhelming evidence and guidance, the availability of assessment and treatment for mental health conditions in HIV clinics and community settings remains limited in many parts of the world.^7^ Many programmes still fall short in adequately addressing mental health treatment. The gap between awareness and action persists, leaving many individuals with mental health needs unsupported.

Here, we highlight several challenges and offer examples of promising practices that showcase how various countries have pragmatically advanced integration.

First, promoting mental health and providing mental health services is often seen as an add-on and not positioned as an essential component of integrated, people-centred prevention and care services. Importantly, consideration must be given not only to the integration of mental health services into HIV prevention, treatment and care, but also the integration of HIV and mental health services more broadly into primary health care. Several low-cost, evidence-based interventions at the health facility and community levels offer proven, cost-effective ways to improve mental health and HIV outcomes as a part of primary health care. For example, community health agents in Brazil play a crucial role in delivering integrated HIV and mental health services through primary care clinics. This model is supported by Brazil’s Family Health Strategy of the Unified Health System, which uses community health agents to provide mental health counselling, antiretroviral therapy adherence support and psychosocial interventions at the community level.

Second, although many psychosocial programmes offer support, including counselling or preventive measures, they often fail to provide treatment for individuals with mental health conditions such as depression, anxiety or post-traumatic stress disorder. Conversely, in Thailand, the Tangerine Community Health Clinic, working closely with the Institute of HIV Research and Innovation, has incorporated mental health services into its inclusive and equitable HIV care for transgender people.^8^

Third, mental health conditions rarely occur in isolation, but programmes are often designed and implemented to diagnose and treat one condition at a time. Programmes that recognize and treat individuals with co-occurring conditions, such as depression and anxiety, tend to show better outcomes than those focusing on just one condition alone.^1^ However, very few integrated mental health and HIV programmes have successfully implemented this approach. A notable exception is in Mozambique, where the Common Elements Treatment Approach, a transdiagnostic evidence-based psychological treatment, has been integrated into routine HIV care in Sofala.^9^ Similarly, ENGAGE is an approach that focuses on training community health workers in Nampula to use a locally validated digital tool called the Electronic Mental Wellness Tool to screen, diagnose and treat common mental and substance use disorders among people living with, at risk of or affected by HIV, and refer those with more serious conditions to trained primary care providers supervised by psychiatric technicians for behavioural and pharmacological evidence-based treatment.^10^ Both are promising practices that recognize and treat co-occurring conditions.

Fourth, many health-care and community settings are not tailored to the specific needs of adolescents, making it difficult for them to engage meaningfully with care. This shortcoming includes limited mental health integration in HIV programmes where virological suppression and antiretroviral therapy adherence are prioritized and mental health needs are considered secondary. In contrast, the Zvandiri programme for adolescents in Zimbabwe offers a compelling example of how peer-led approaches can improve mental health and HIV outcomes through emotional support, adherence counselling and mental health screening. The Zvandiri programme improves antiretroviral therapy adherence and empowers young people to take an active role in their own care, a critical factor in sustainable health outcomes.^11^ The programme has been implemented in 13 African countries, focusing on community-based interventions that leverage local resources and peer support systems, ensuring that children and caregivers are not overlooked.

With a shrinking fiscal space, many funders are regressing to the essentials of HIV programming, that is, lifesaving services. However, evidence shows that integrating mental health care into HIV programmes is highly cost-effective, improving access to HIV services, retention in HIV care and overall health outcomes.^1^^–^^3^^,^^12^^,^^13^ These mutual benefits reduce long-term health-care costs and enhance the efficiency of HIV prevention and care, making such integration a smart investment even in resource-constrained settings.^12^^,^^13^ Mental health must not be treated as a secondary consideration but remain as an integral part of the HIV response. Protecting the return on investment in HIV prevention, screening, diagnosis and treatment requires a deliberate focus on enhancing HIV outcomes, something that is considerably compromised by unaddressed mental health conditions. Mental health integration is not only cost-effective but essential to safeguarding the 18.7 billion United States dollars (US$) already invested in the HIV response in low- and middle-income countries in 2024 alone. Economic modelling suggests that for every US$ 1.00 invested in treating common mental disorders, up to US$ 5.70 is saved in economic cost and health returns.^14^ For HIV, savings are estimated at US$ 6.40.^15^

Importantly, integrating mental health into HIV programmes should also be positioned as part of a broader, system-wide effort to strengthen primary health care. By embedding mental health services within primary health care, health systems can respond more holistically to the needs of individuals, especially in high HIV-burden settings where primary care is often the first and most frequent point of contact. This approach reduces fragmentation, increases efficiency, addresses stigma and builds resilient health and community systems capable of managing co-occurring conditions, thus maximizing the impact of HIV investments while contributing to the wider goals of universal health coverage. To achieve this, we need stronger partnerships across different actors to translate global guidance into reality through the implementation of practical, context-specific solutions that acknowledge the underlying system weaknesses. This approach means engaging diverse stakeholders, including governments, civil society, health providers and affected communities, to co-design and co-deliver these solutions. Additionally, prioritizing mental health funding within and beyond HIV programmes is essential. Clear metrics to measure progress on integration and its impact on HIV outcomes should be established. Substantial implementation research is still needed to assess integrated care, its application, performance and impact in various settings and populations, particularly in low- and middle-income countries. Collaborative learning for facilitating knowledge sharing, problem-solving and promoting evidence-based practices will accelerate implementation and ensure sustainability.

We call on national governments, donors and implementers to commit to this agenda by allocating resources, fostering accountability mechanisms and scaling up proven models of integrated care. These efforts will unite HIV, mental health, primary care and broader public health communities, globally and locally, helping to build more effective, integrated programmes that improve the quality of life for individuals and families affected by both HIV and mental health conditions. By acting now, we can close the gap, address longstanding disparities and create a future where mental health is a foundational pillar of HIV prevention, treatment and care.
